# Supplementary material for: Prolonged tau clearance and stress vulnerability rescue by pharmacological activation of autophagy in tauopathy neurons
Source: Nat Commun. 2020 Jun 26;11:3258. doi: 10.1038/s41467-020-16984-1 (PMC7320012; doi:10.1038/s41467-020-16984-1)
Supplement: Supplementary file 1 — Supplementary Information [file 41467_2020_16984_MOESM1_ESM.pdf]

Prolonged tau clearance and stress vulnerability rescue by pharmacological activation of autophagy in tauopathy neurons

Silva *et al*.

Supplementary Information

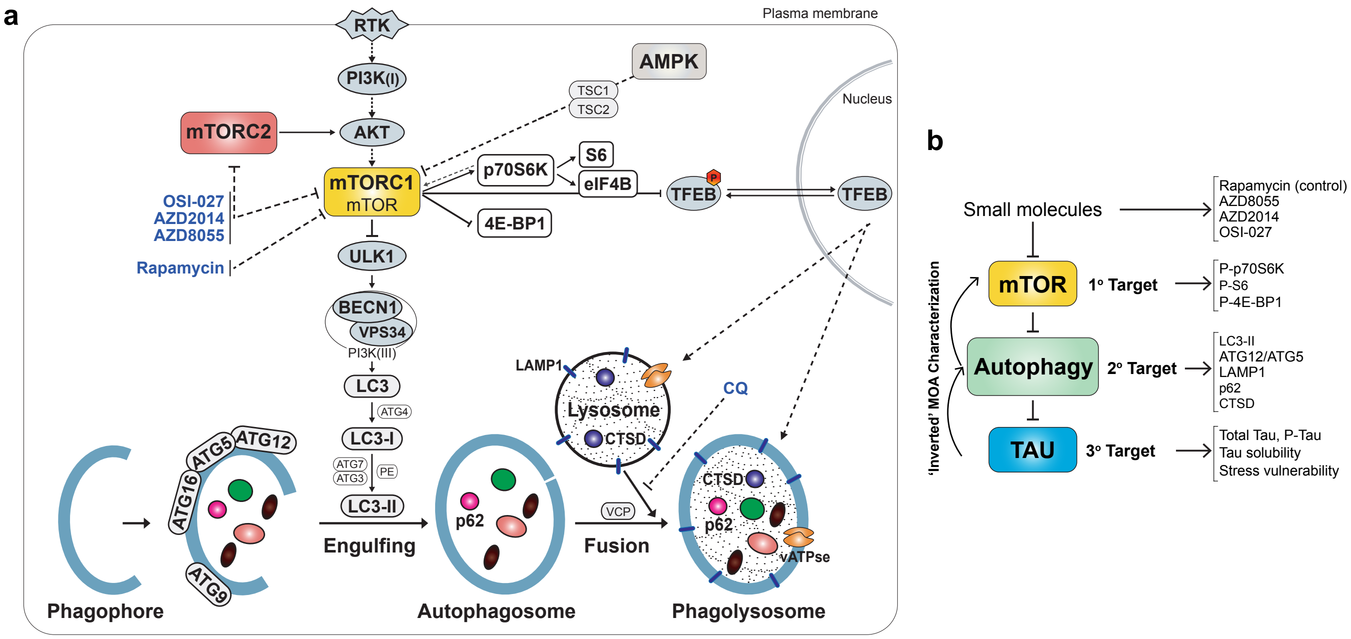

Supplementary Fig. 1. a Simplified schematic of mTOR complexes 1 and 2 (mTORC1/2) regulation of autophagy, and predicted molecular targets for OSI-027, AZD2014, AZD8055 and rapamycin. Dotted lines indicate predicted or indirect interaction/regulation. **b** Overview of the proposed mechanism-of-action (MOA) for the mTOR inhibitors (mTORi) under investigation, regarding primary and downstream targets, and the molecular markers measured to test effect on each target.

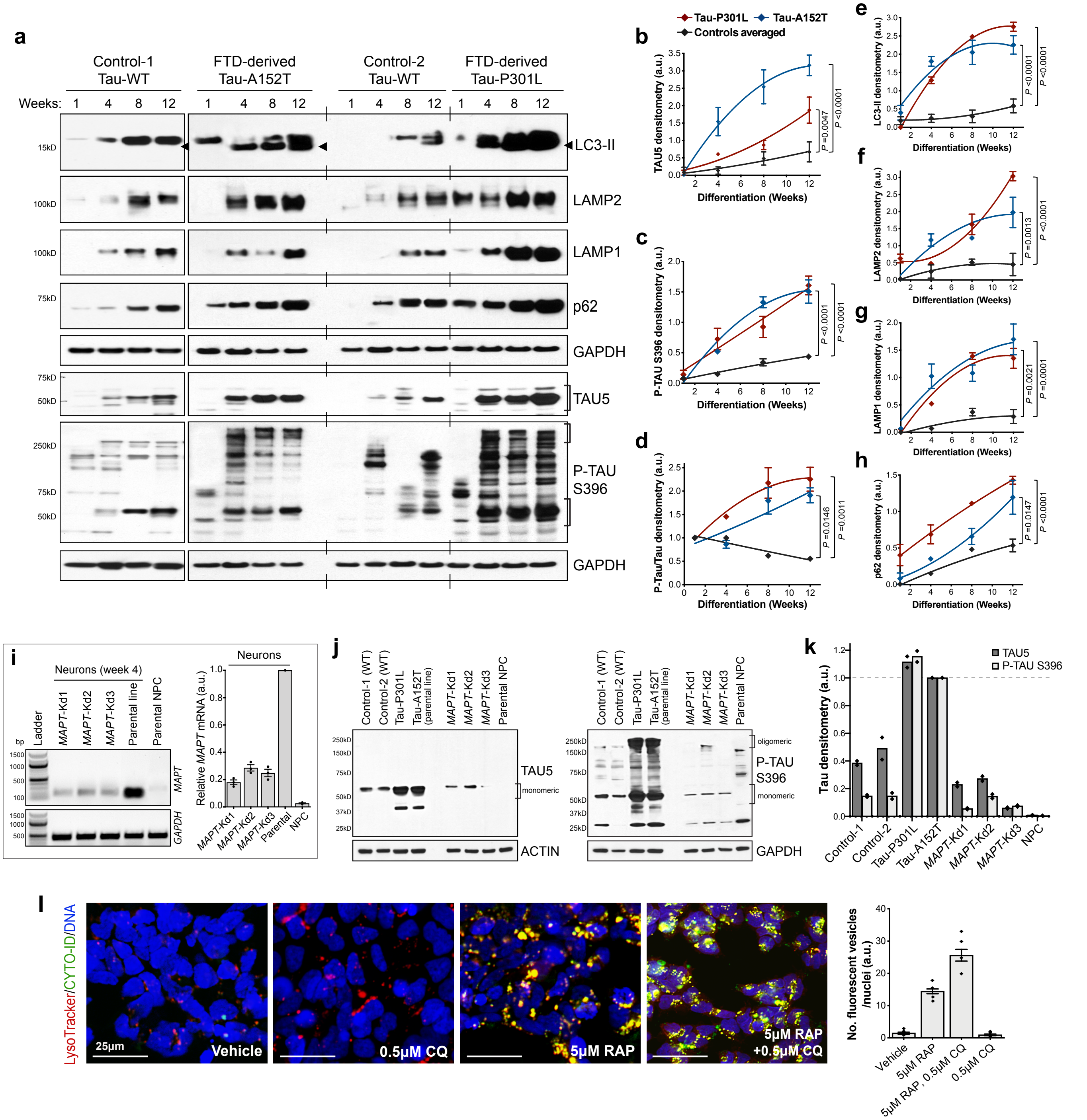

Supplementary Fig. 2. Tau and autophagy markers in tauopathy neuronal cell models. **a** Time-course analysis of autophagy (LC3-II, LAMP2, LAMP1, p62), total tau (TAU5) and P-tau^{S396} levels, in non-mutant Control-1 (8330-8-RC1) and Control-2 (MGH2069-RC1), tau-A152T (FTD19-L5-RC6) and tau-P301L (MGH2046-RC1) neurons differentiated for one to 12 weeks. Representative western blots of $N = 3$ biological replicates. **b - h** Graph data points represent mean densitometry ± SEM of $N = 3$. Statistical significance was calculated with two-way ANOVA with post hoc Dunnett's multiple comparisons test relative to controls (P-values are indicated in each graph). **i** *MAPT* mRNA levels by RT-PCR and gel electrophoresis analysis, comparing the parental line tau-A152T (FTD19-L5-RC6) neurons and NPCs with the respective CRISPR/Cas9-engineered *MAPT* knockdown (*MAPT*-Kd) lines 1-3. Graph bars represent mean intensity of each band, normalized to *GAPDH* and relative to levels in the parental neuronal sample ± SD, and black circles represent data points for $N = 3$. **j** Analysis of TAU5 and P-tau^{S396} antibodies band patterns in non-mutant Control-1 (8330-8-RC1), Control-2 (MGH2069-RC1), tau-A152T (FTD19-L5-RC6) and tau-P301L (MGH2046-RC1) 4-week differentiated neurons, relative to *MAPT*-Kd neurons and tau-A152T NPCs (no tau expression control). Tau and P-tau bands of higher specificity are indicated within brackets and quantified in **k.** Bars represent

mean tau densitometry relative to A152T neurons and diamond dots represent individual data points for N = 2 biological replicates. **l** Control-1 (8330-8-RC1) NPCs treated with rapamycin (RAP) and chloroquine (QC), followed by LysoTracker and CYTO-ID staining and imaging. Co-treatment was used to exacerbate autophagy vesicles accumulation for imaging (RAP+CQ). Micrographs representative of N = 4. Graph shows quantification of LysoTracker^{+}/CYTO-ID^{+} vesicles per nuclei averaged ± SD, and diamond-dots represent individual data points for N = 4 biological replicates. Source data are provided as a Source Data file.

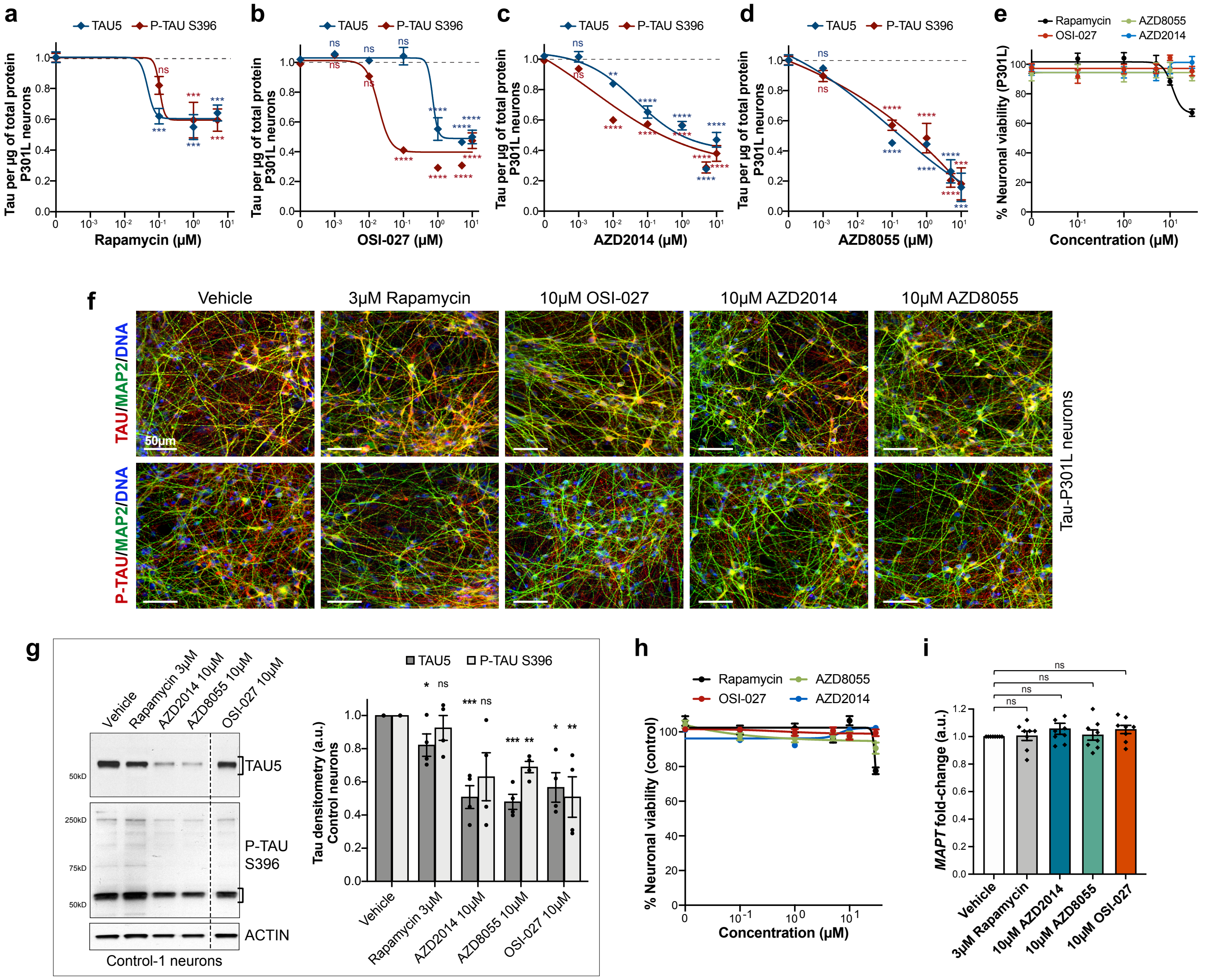

Supplementary Fig. 3. Effect of mTORi on tau-P301L and non-mutant Control-1 neurons. **a - d** Compound dose-curves for total tau (TAU5) and P-tau^{S396} in P301L neurons (MGH2046-RC1 6-week differentiated), by ELISA. Data points represent mean tau per µg of total protein, relative to vehicle-treated ± SEM, for $N = 4$ biological replicates. Statistical significance was calculated using two-way ANOVA with post hoc Dunnett's multiple comparisons test relative to vehicle ($^{ns}P > 0.05$, $^{**}P \leq 0.01$, $^{***}P \leq 0.001$, $^{****}P \leq 0.0001$). **e** Mean % viability dose-curves for 24h-treated P301L neurons ± SEM, for $N = 4$ biological replicates. **f** IF of 8-week differentiated P301L (MGH2046-RC1) neurons, treated with mTORi for 24 hrs, with total tau (K9JA) and P-tau$^{S396/S404}$ (PHF-1) antibodies in red, and neuronal marker MAP2 in green. Representative micrographs of $N = 3$. **g** Total tau (TAU5) and P-tau^{S396} levels upon 24 hrs compound treatment of 6-week differentiated Control-1 (8330-8-RC1) neurons. Representative western blot is shown on the left (samples were run on the same gel and the image was cropped at the dotted line only for the purpose of this figure). On the right, graph bars represent mean tau densitometry (a.u.) relative to vehicle-treated samples ± SEM, and black circles correspond to individual data points for $N = 4$ biological replicates. Statistical significance was calculated by a two-tailed unpaired t-test ($^{ns}P > 0.05$, $^{*}P \leq 0.05$, $^{**}P \leq 0.01$, $^{***}P \leq 0.001$). **h** Viability dose-curves for Control-1 (8330-8-RC1) neurons upon 24 hrs treatment ($N = 4$). **i** qRT-PCR measurement of *MAPT* mRNA levels in 10-week differentiated A152T neurons treated for 24 hrs with mTORi at 3µM (rapamycin) or 10µM (OSI-027, AZD2014, AZD8055). Bars represent mean fold-change ± SEM and diamond dots represent individual data points for $N = 2$ biological replicates (with 4 technical replicates). Statistical analysis was done using two-tailed unpaired t-test ($^{ns}P > 0.05$). Source data are provided as a Source Data file.

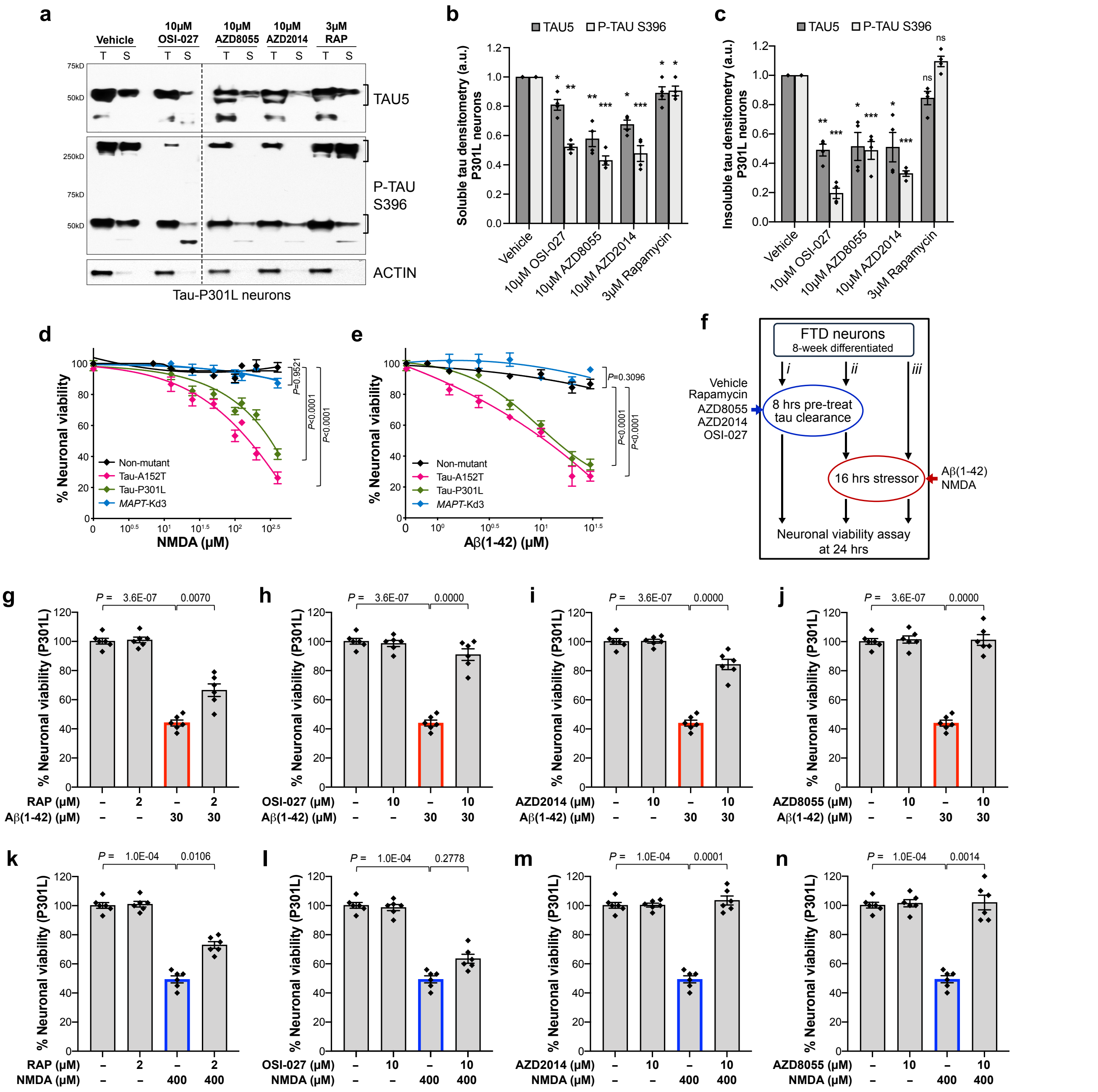

Supplementary Fig. 4. Rescue of tau phenotypes in 8-week differentiated P301L (MGH2046-RC1) neurons. **a - c** Protein fractionation based on detergent solubility (T, Triton-X100 fraction and S, SDS fraction) after 24 hrs treatment. Representative western blot and mean densitometry (bands within brackets) ± SEM for detergent-soluble (**b**) and detergent-insoluble (**c**) total tau (TAU5) and P-tau^{S396}. Samples were run on the same blot (**a**) and the image was cropped at the dotted line only for the purpose of this figure. Diamond dots (**b**, **c**) represent individual data points for $N = 4$ biological replicates. Statistical significance was calculated by a two-tailed unpaired *t*-test ($^{ns}P > 0.05$, $*P \leq 0.05$, $**P \leq 0.01$, $***P \leq 0.001$, $****P \leq 0.0001$). **d - e** NMDA and Aβ(1-42) concentration- and genotype-dependent induced loss of neuronal viability. Data points represent mean % viability ± SEM for $N = 4$ biological replicates. Statistical significance was calculated using two-way ANOVA relative to Control-1 ($^{ns}P > 0.05$, $****P < 0.0001$, with exact *P*-values indicated on each graph). **f** Assay for testing rescue of stress vulnerability to NMDA or Aβ(1-42) (*iii,* 16 hrs exposure) by pre-treating neurons with mTORi for 8 hrs (*ii*). Effect of mTORi alone was also tested (*i*). **g - n** Rescue of P301L neuronal vulnerability to Aβ(1-42) (30μM, red) and NMDA (400μM, blue), by treatment with (**g, k**) 3μM rapamycin, (**h, l**) 10μM OSI-027, (**i, m**) 10μM AZD2014 and (**j, n**) 10μM AZD8055. Bars represent mean % viability relative to vehicle ± SEM, and diamond-dots represent individual data points for $N = 3$ biological replicates (and 2 technical replicates). Statistical significance was calculated with a two-tailed unpaired *t*-test, and *P*-values are indicated (same significance intervals apply $^{ns}P > 0.05$, $*P \leq 0.05$, $**P \leq 0.01$, $***P \leq 0.001$, $****P \leq 0.0001$). Source data are provided as a Source Data file.

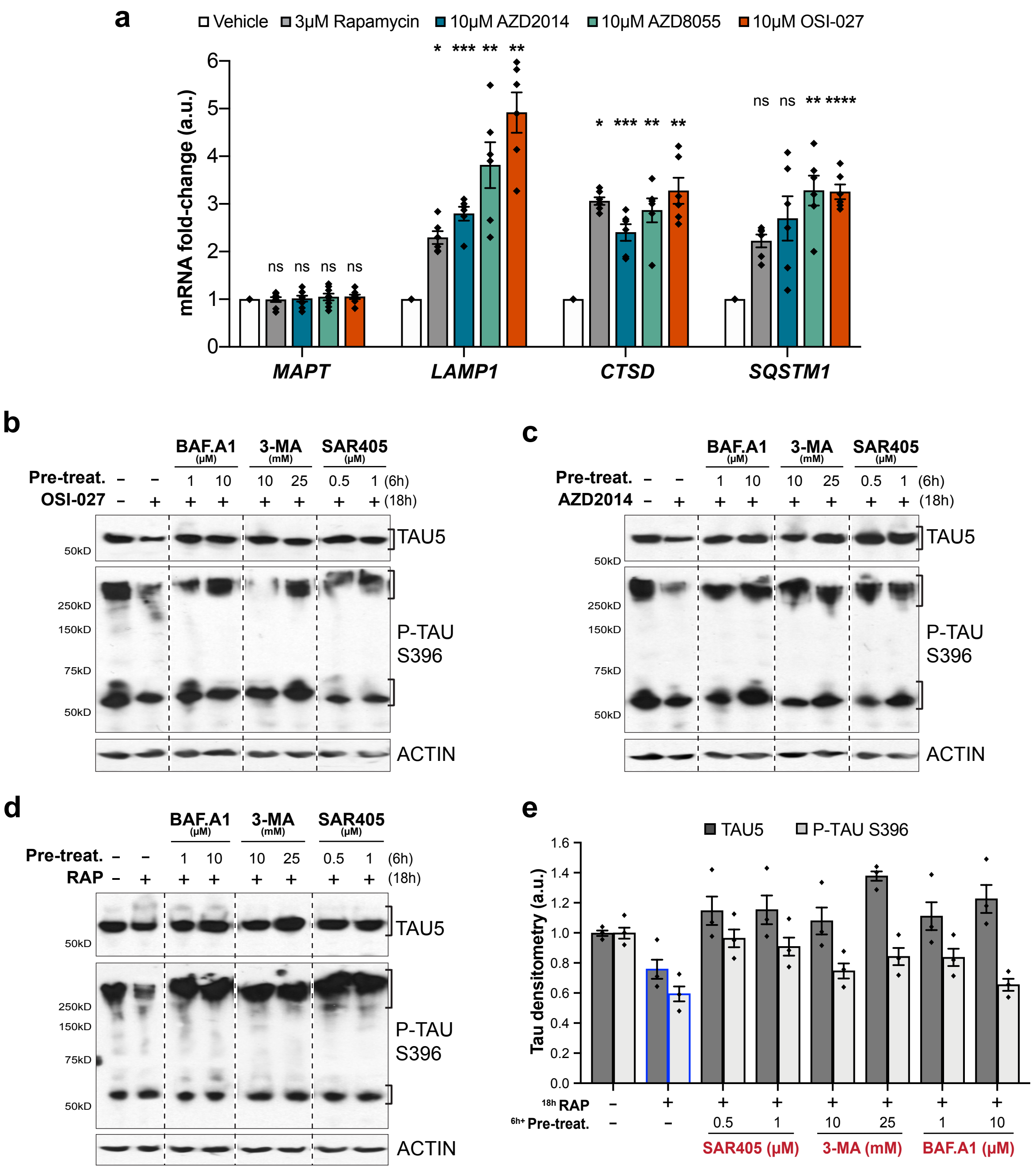

Supplementary Fig. 5. Effect of mTORi on tau-A152T neurons is autophagy-dependent. **a** mRNA level of *LAMP1*, *CTSD* and *p62*, as well as *MAPT*, measured by qRT-PCR in 10-week differentiated A152T (FTD19-L5-RC6) neurons treated for 24 hrs with mTORi or vehicle alone. Graph bars represent mean fold-change ± SD and diamond-dots represent individual data points for $N = 2$ biological replicates (and 3 technical replicates). Statistical significance was calculated with two-tailed unpaired *t*-test, based on ΔC_T values relative to vehicle ($^{ns}P > 0.05$, $*P \leq 0.05$, $**P \leq 0.01$, $***P \leq 0.001$, $****P \leq 0.0001$). **b - e** Tau downregulation dependence on autophagy was tested with pre-treatment of A152T (FTD19-L5-RC6) neurons with SAR405, 3-MA or BAF.A1 for 6 hrs, followed by 10μM OSI-027 (**b**, see Fig. 4j), 10μM AZD2014 (**c**, see Fig. 4k) or 3μM rapamycin (**d, e**), for a total of 24 hrs. Representative western blots for $N = 3$ biological replicates are shown (**b - d** samples were run on the same gel and the images were cropped at the dotted line only for the purpose of this figure). **e** Graph bars represent mean tau densitometry ± SEM, and diamond-dots represent individual data points for $N = 3$ biological replicates. Source data are provided as a Source Data file.

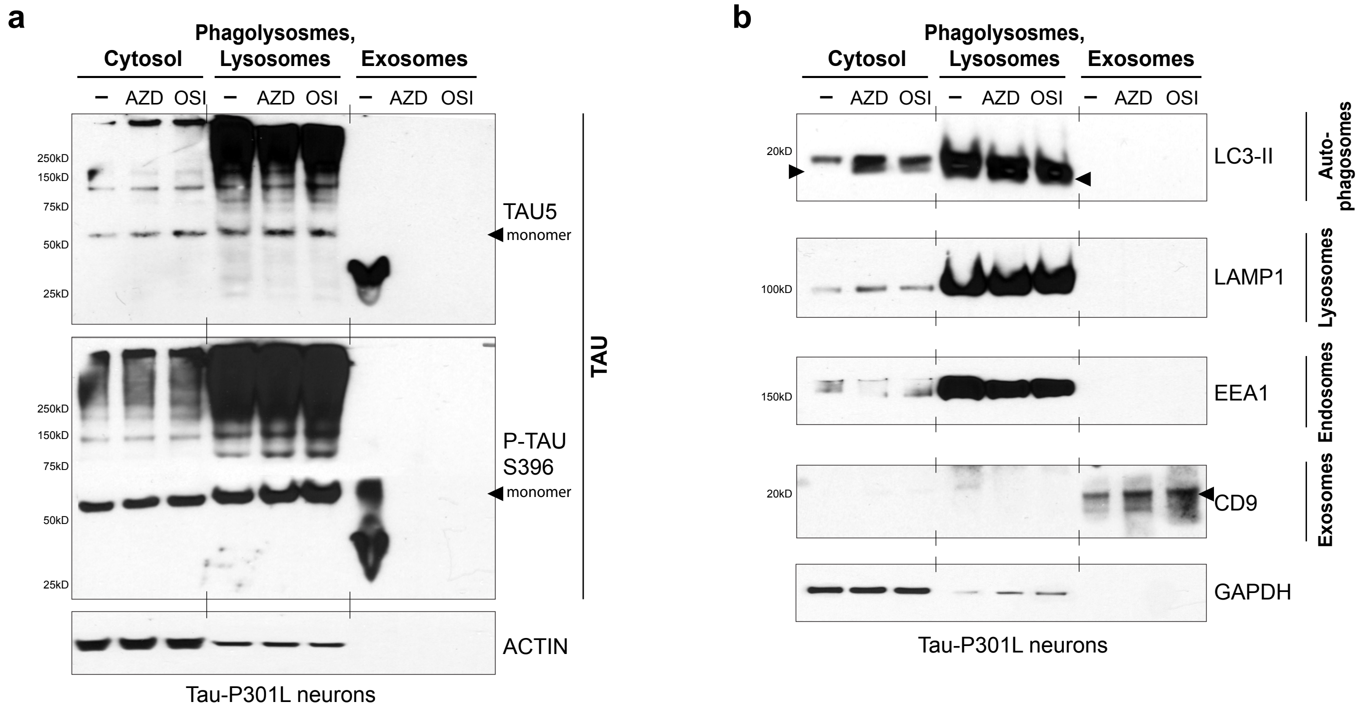

Supplementary Fig. 6. Demonstration of tau recruitment into lysosomes/phagolysosomes in 6-week differentiated P301L (MGH2046-RC1) neurons, upon 8 hrs treatment with 10µM AZD2014 (AZD), 10µM OSI-027 (OSI) or vehicle-alone (–). **a** Cellular fractionation and western blot of tau in cytosol, phagolysosomes/lysosomes and exosomes fractions. **b** Fraction-specific markers were immunoprobed as controls. Blots are representative of $N = 2$ biological replicates.

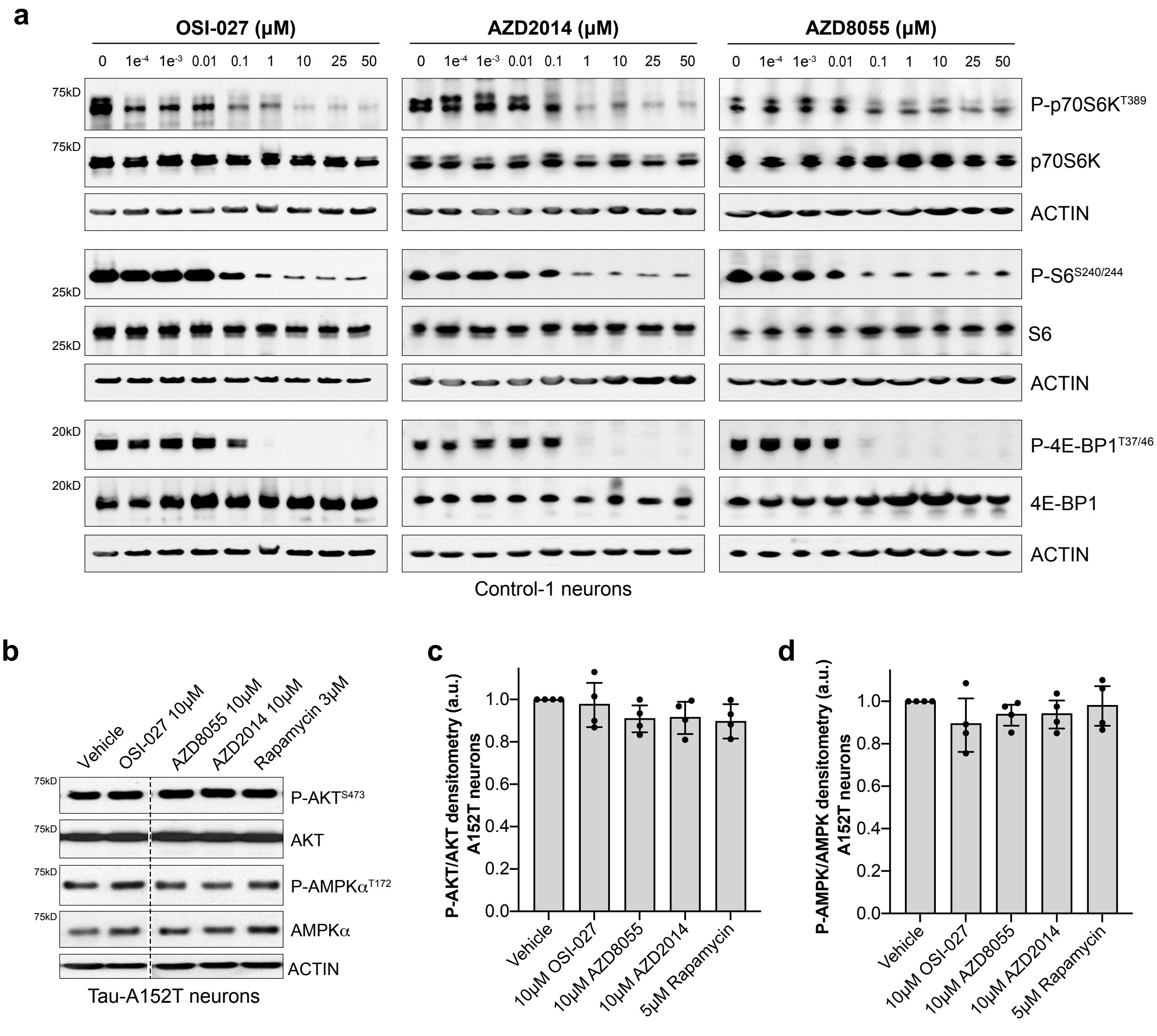

Supplementary Fig. 7. Compound effect on mTORC1, mTORC2 and AMPK substrates' phosphorylation. **a** Dose-effect of OSI-027, AZD2014 and AZD8055 on phosphorylation of mTORC1 substrates p70S6K, S6 and 4E-BP1. Western blot analysis of 6-week differentiated Control-1 (8330-8-RC1) neurons treated for 24 hrs with doses between 0.1nM-50µM (representative blot of $N = 2$, quantification shown in Fig. 6b-d). **b** Effect of mTORi on P-AKT^{S473} (mTORC2 substrate) and P-AMPK^{T172} (AMPK activity) after 24 hrs treatment of A152T (FTD19-L5-RC6) neurons, by western blot analysis (samples were run on the same gel and the image was cropped at the dotted line only for the purpose of this figure). Representative blot image for $N = 2$ biological replicates and 2 technical replicates. **c, d** Graph bars represent mean densitometry of each phospho-marker normalized to total levels and relative to vehicle-treated samples ± SD. Black circles represent individual data points for $N = 2$ biological replicates and 2 technical replicates per sample set. Source data are provided as a Source Data file.

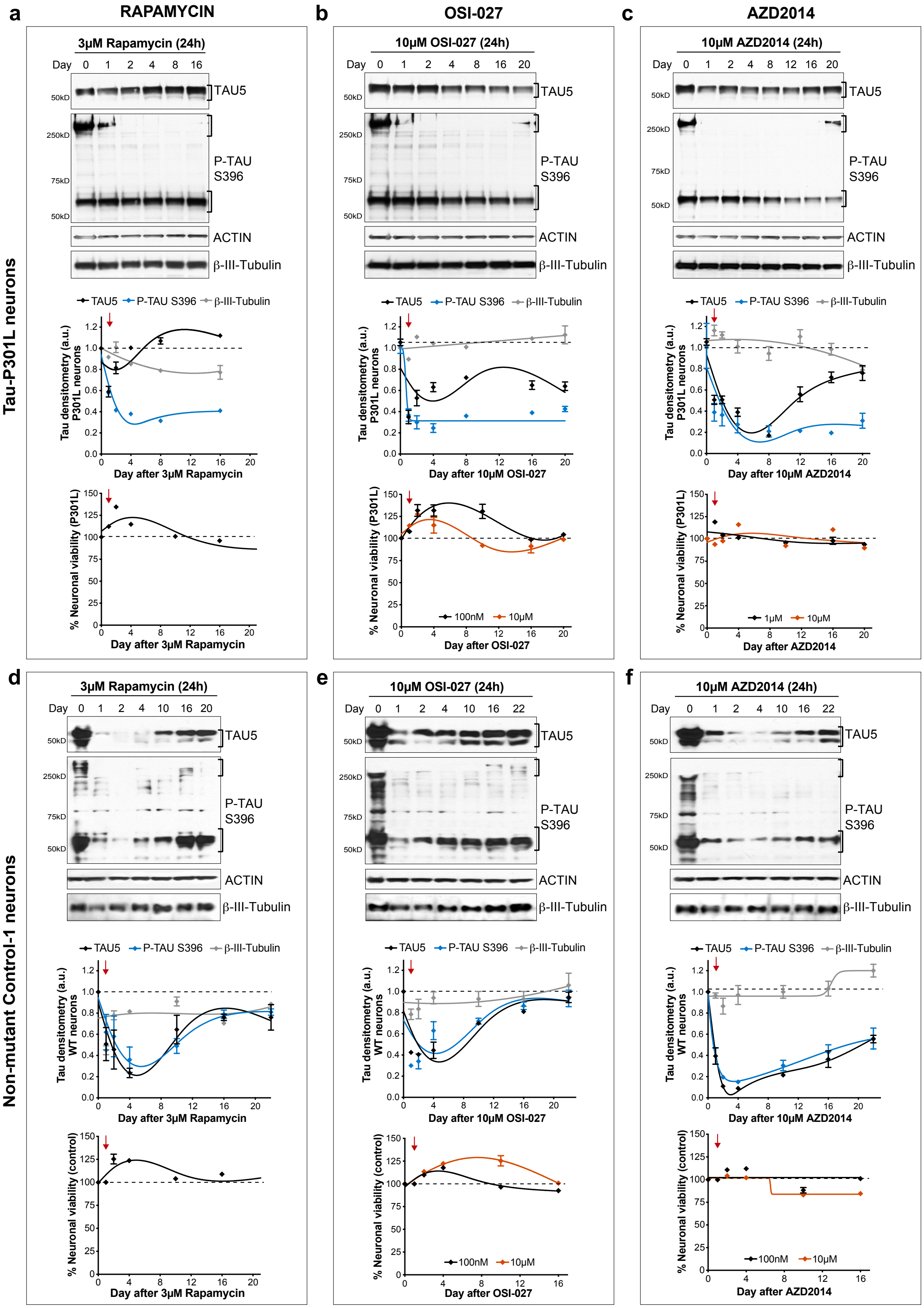

Supplementary Fig. 8. Time-course measure of total tau (TAU5), P-tau^{S396} and β-III-tubulin protein levels upon 24 hrs mTORi treatment of P301L neurons (**a - c,** MGH2046-RC1) and non-mutant Control-1 neurons (**d - f,** 8330-8-RC1), followed by compound washout (red arrow). Representative western blots, mean densitometry and neuronal viability are shown over the course of 20 days. Data points represent mean values relative to vehicle ± SEM for $N = 3$ biological replicates. Source data are provided as a Source Data file.

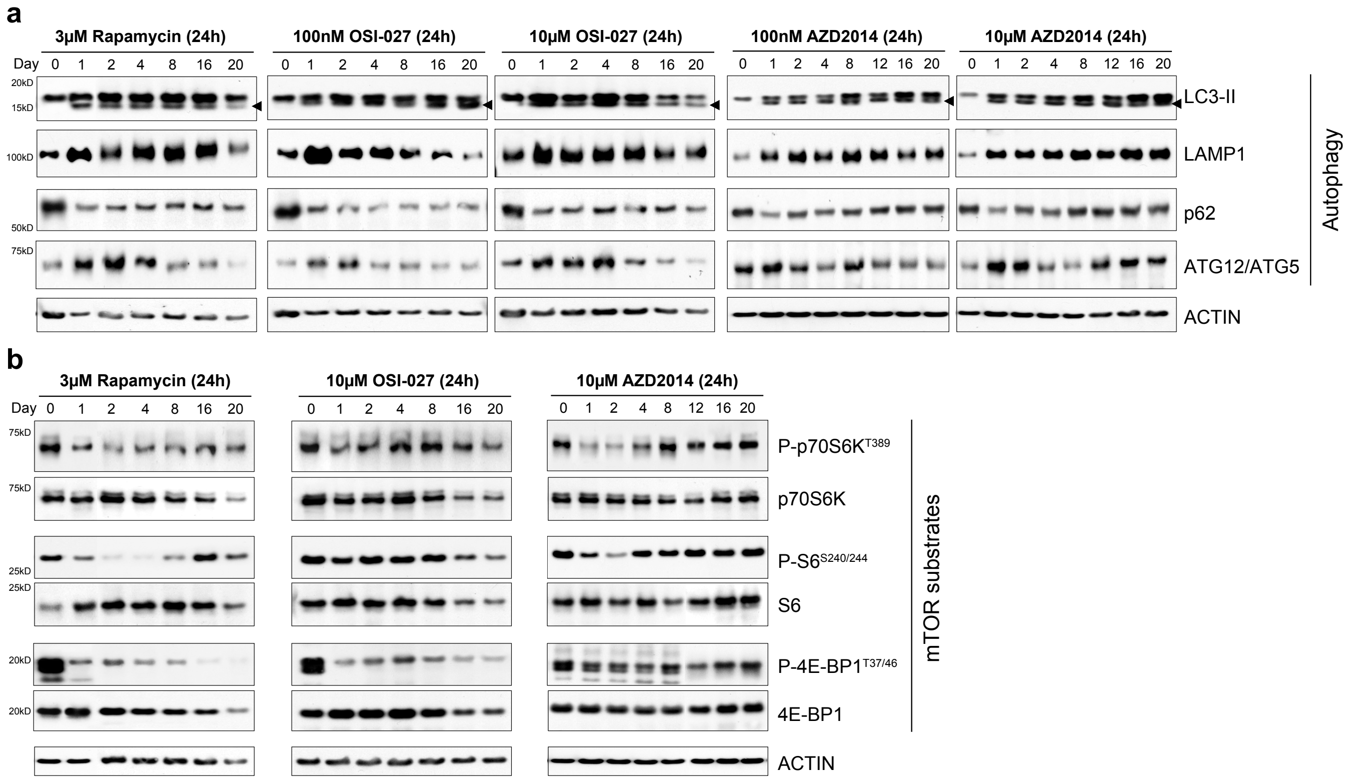

c

Treatment (hrs)	AZD2014 (μM)	Intracellular AZD2014 (μM)	Intracellular AZD2014 (pmol/mg protein)	Intracellular % Free AZD2014	Extracellular AZD2014 (μM)
24	1	0.4 ± 0.15 (40%)	109 ± 27.7	-	-
24	10	2.0 ± 0.58 (20%)	513 ± 67.3	4.5 ± 0.03	7.4 ± 0.10

Supplementary Fig. 9. Time-course analysis of mTORi effect on autophagy and mTORC1 activity up to 20 days post-treatment of A152T (FTD19-L5-RC6) neurons. **a, b** Representative western blots corresponding to the graphs in Fig. 8c-q. **a** Western blots of autophagy markers LC3-II, ATG12, LAMP1 and p62, representative of $N = 3$ biological replicates. **b** Western blots of mTORC1 substrates' phosphorylation (p70S6K, S6, 4E-BP1), representative of $N = 2$ biological replicates. **c** Analysis of cell lysates and extracellular media from Control-1 (8330-8-RC1) neurons treated with 1μM or 10μM AZD2014. Micro-dialysis and mass spectrometry evidence of compound permeability and intracellular AZD2014 concentration ± SD, at 4 hrs and 24 hrs of treatment. Data corresponds to $N = 3$ biological replicates. Source data are provided as a Source Data file.

Supplementary Table 1 Autophagy modulators that rescued tau phenotypes in FTD patient-derived neuronal cell models.

Compound	Molecule	Commercial Information	Target	*In vitro* data	Clinical
**Rapamycin** (Sirolimus Rapamune)	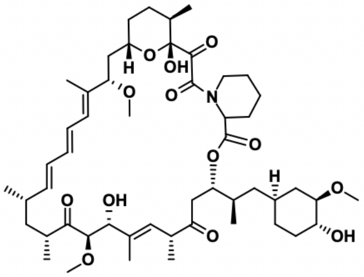	Fisher Scientific NC9362949	Selective mTORC1 inhibitor	IC_{50} 0.1 nM	FDA approved 1999
**OSI-027**	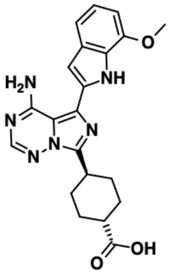	Selleck S2624	ATP-competitive, mTOR inhibitor (mTORC1/2)	$IC_{50,\ mTORC1}$ 22 nM $IC_{50,\ mTORC2}$ 65 nM	Investigational (cancer)
**AZD2014** (Vistusertib)	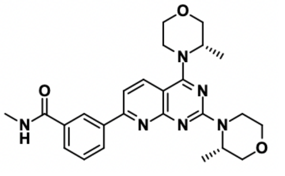	Selleck S2783	ATP-competitive, mTOR inhibitor (mTORC1/2)	IC_{50} 2.8 nM	Investigational (cancer)
**AZD8055**	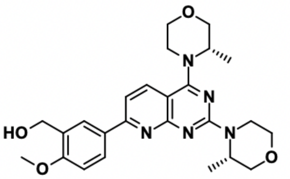	Cellagen Technology C2955-5	ATP-competitive, mTOR inhibitor (mTORC1/2)	IC_{50} 0.8 nM	Investigational (cancer)

Supplementary Table 2 Antibodies and compounds commercial information.

Reagent type	Designation	Source (clone and dilution for antibodies)
Antibody, western blot	TAU5	Invitrogen AHB0042 (1:1000)
Antibody, western blot	P-tau^{S396}	Invitrogen 44752G (1:1000)
Antibody, western blot	LC3	Cell Signaling Technology 3868, clone D11 (1:1000)
Antibody, western blot	LAMP2	Abcam ab18528 (1:1000)
Antibody, western blot	LAMP1	Cell Signaling Technology 9091, clone D2D11 (1:1000)
Antibody, western blot	p62	Enzo Lifesciences BML-PW9860 (1:2000)
Antibody, western blot	P-p62^{S403}	GeneTex GTX128171 (1:500)
Antibody, western blot	ATG12	Cell Signaling Technology 2010 (1:500)
Antibody, western blot	CTSD	Cell Signaling Technology 2284 (1:1000)
Antibody, western blot	mTOR	Cell Signaling Technology 2983T, clone 7C10 (1:1000)
Antibody, western blot	P-mTOR^{S2448}	Cell Signaling Technology 5536T, clone D9C2 (1:1000)
Antibody, western blot	p70S6K	Cell Signaling Technology 9202 (1:1000)
Antibody, western blot	P-p70S6K^{T389}	Cell Signaling Technology 9205S (1:1000)
Antibody, western blot	S6	Cell Signaling Technology 2317, clone 54D2 (1:500)
Antibody, western blot	P-S6$^{S240/244}$	Cell Signaling Technology 2215 (1:1000)
Antibody, western blot	4E-BP1	Cell Signaling Technology 9644S (1:1000)
Antibody, western blot	P-4E-BP1$^{T37/46}$	Cell Signaling Technology 9451S (1:1000)
Antibody, western blot	BECN1	Cell Signaling Technology 4122S, clone 2A4 (1:1000)
Antibody, western blot	P-BECN1^{S15}	Cell Signaling Technology 84966S, clone D4B7R (1:1000)
Antibody, western blot	AKT	Cell Signaling Technology 9272 (1:1000)
Antibody, western blot	P-AKT^{S473}	Cell Signaling Technology 4060, clone D9E (1:1000)
Antibody, western blot	AMPKα	Cell Signaling Technology 5831, clone D5A2 (1:1000)
Antibody, western blot	P- AMPKα^{T172}	Cell Signaling Technology 2535, clone 40H9 (1:1000)
Antibody, western blot	HSP90	Cell Signaling Technology 4877, clone C45G5 (1:1000)
Antibody, western blot	COX IV	Cell Signaling Technology 4850S, clone 3E11 (1:1000)
Antibody, western blot	HDAC2	Abcam ab7029 (1:5000)
Antibody, western blot	EEA1	Cell Signaling Technology 3288S, clone C45B10 (1:1000)
Antibody, western blot	RAB5	Cell Signaling Technology 2143S (1:1000)
Antibody, western blot	CD9	Cell Signaling Technology 13403S, clone D3H4P (1:500)
Antibody, western blot	CD81	Santa Cruz sc-23962, clone 5A6 (1:250)
Antibody, western blot	β-III-Tubulin	Sigma T-8660 (1:5000)
Antibody, western blot	GAPDH	Abcam ab8245, clone 6C5 (1:5000)
Antibody, western blot	β-Actin	Sigma A1978, clone AC-15 (1:10,000)
Antibody, IF	K9JA	Agilent A002401-2 (1:1000)
Antibody, IF	PHF-1	Dr. Peter Davies (Albert Einstein College of Medicine) (1:1000)
Antibody, IF	MAP2	Millipore Sigma AB5543 (1:1000)
Antibody, IF	Hoechst 33342	Thermo Fisher Scientific 62249 (1:2500)
Secondary antibody (WB)	Anti-mouse IgG HRP-linked	Cell Signaling Technology 7076V (1:4000)
Secondary antibody (WB)	Anti-rabbit IgG HRP-linked	Cell Signaling Technology 7074S (1:4000)
Secondary antibody (IF)	Goat anti-chicken IgG Alexa Fluor 488	Life Technologies A11039 (1:500)
Secondary antibody (IF)	Goat anti-chicken IgY Alexa Fluor 594	Life Technologies A11042 (1:500)
Secondary antibody (IF)	Goat anti-rabbit IgG Alexa Fluor 594	Invitrogen A11012 (1:500)
Secondary antibody (IF)	Goat anti-mouse IgG Alexa Fluor 488	Invitrogen A11029 (1:500)
Secondary antibody (IF)	Goat anti-mouse IgG Alexa Fluor 594	Life Technologies A11032 (1:500)
Compound	Rapamycin	Fisher Scientific NC9362949
Compound	3-Methyladenine	Sigma M9281
Compound	Chloroquine	Sigma C6628
Compound	OSI-027	Selleck S2624
Compound	AZD2014	Selleck S2783
Compound	AZD8055	Cellagen Technology C2955
Compound	SAR405	MedChemExpress HY-12481
Compound	Bafilomycin A1	Enzo Life Sciences BML-CM110
Compound	MHY-1485	MedChemExpress HY-B0795
Compound, stressor	Aβ(1-42)	Abcam ab120481
Compound, stressor	NMDA	Selleck S7072
