# Supplementary material for: Prolonged tau clearance and stress vulnerability rescue by pharmacological activation of autophagy in tauopathy neurons
Source: Nat Commun. 2020 Jun 26;11:3258. doi: 10.1038/s41467-020-16984-1 (PMC7320012; doi:10.1038/s41467-020-16984-1)
Supplement: Supplementary file 3 — Reporting summary [file 41467_2020_16984_MOESM3_ESM.pdf]

nature research

Corresponding author(s): Stephen J. Haggarty

Last updated by author(s): 2020/05/05

Reporting Summary

Nature Research wishes to improve the reproducibility of the work that we publish. This form provides structure for consistency and transparency in reporting. For further information on Nature Research policies, seeAuthors & Referees and theEditorial Policy Checklist .

Statistics

For all statistical analyses, confirm that the following items are present in the figure legend, table legend, main text, or Methods section.

n/a	Confirmed	
☐	☒	The exact sample size (*n*) for each experimental group/condition, given as a discrete number and unit of measurement
☐	☒	A statement on whether measurements were taken from distinct samples or whether the same sample was measured repeatedly
☐	☒	The statistical test(s) used AND whether they are one- or two-sided<br>*Only common tests should be described solely by name; describe more complex techniques in the Methods section.*
☒	☐	A description of all covariates tested
☐	☒	A description of any assumptions or corrections, such as tests of normality and adjustment for multiple comparisons
☐	☒	A full description of the statistical parameters including central tendency (e.g. means) or other basic estimates (e.g. regression coefficient) AND variation (e.g. standard deviation) or associated estimates of uncertainty (e.g. confidence intervals)
☒	☐	For null hypothesis testing, the test statistic (e.g. *F*, *t*, *r*) with confidence intervals, effect sizes, degrees of freedom and *P* value noted<br>*Give P values as exact values whenever suitable.*
☒	☐	For Bayesian analysis, information on the choice of priors and Markov chain Monte Carlo settings
☒	☐	For hierarchical and complex designs, identification of the appropriate level for tests and full reporting of outcomes
☒	☐	Estimates of effect sizes (e.g. Cohen's *d*, Pearson's *r*), indicating how they were calculated

Our web collection on statistics for biologists contains articles on many of the points above.

Software and code

Policy information about availability of computer code

Data collection	Data collection described in Methods section. No custom software was used.
Data analysis	Data analysis is described in the Methods section. Lysotracker/CYTOID screen images were analyzed with CellProfiler version 2.2.0, a free open-source software, and the analysis pipeline was adapted from CellProfiler.com (cell/particle stained objects counting). Western blot images and micrograph images were assembled in Adobe Photoshop 2020 version 21.1.2. Western blot densitometry was determined using the Histogram function of Adobe Photoshop 2020 version 21.1.2. qRT-PCR data was analyzed in Microsoft Excel version 16.36. Graphs were plotted using GraphPad Prism 8 version 8.4.2. Statistical analysis was done in GraphPad Prism 8 or Microsoft Excel version 16.36. Software used for mass spectrometry quantitation of intracellular/free-compound AZD2014 was Agilent MassHunter version B.07.01 SP2. For in vivo work, the LC-MS/MS data acquisition and processing was done using the Analyst software (AB Sciex, USA) version 1.5.2. Free brain concentrations, free plasma concentrations and the coefficient of partition of the unbound (or free) brain concentrations to the unbound (or free) plasma concentrations (Kpu,u) were calculated using Microsoft Excel 2016.

For manuscripts utilizing custom algorithms or software that are central to the research but not yet described in published literature, software must be made available to editors/reviewers. We strongly encourage code deposition in a community repository (e.g. GitHub). See the Nature Research guidelines for submitting code & software for further information.

Data

Policy information about availability of data

All manuscripts must include a data availability statement. This statement should provide the following information, where applicable:

- Accession codes, unique identifiers, or web links for publicly available datasets
- A list of figures that have associated raw data
- A description of any restrictions on data availability

Data supporting the findings of this study are available within the article and its Supplementary Figures.
The source data underlying Fig. 1-4, Fig. 6-8, Supplementary Figs. 2-5 and Supplementary Figs. 7-9 are provided as a Source Data file. Additional details on datasets

and protocols that support the findings of this study will be made available by the corresponding author upon reasonable request.

Field-specific reporting

Please select the one below that is the best fit for your research. If you are not sure, read the appropriate sections before making your selection.

- [x] Life sciences
- [ ] Behavioural & social sciences
- [ ] Ecological, evolutionary & environmental sciences

For a reference copy of the document with all sections, see nature.com/documents/nr-reporting-summary-flat.pdf

Life sciences study design

All studies must disclose on these points even when the disclosure is negative.

Sample size	In general, no calculations were done to determine sample size. Sample size was determined based on standards for experimental stem cell biology and animal studies, attempting to have a minimum of N = 3 biological replicates with sufficient reproducibility. Exceptions included experiments where an extraordinary large sample size was required, a limiting factor for iPSC-derived neuronal cultures. Regarding cell numbers per experiment, the parameters employed were such that healthy cell cultures were maintained and studied, while providing enough material for protein and mRNA expression levels analysis. NPCs/neurons culture density for each experiment is included in the Methods. In experiments with NPCs and differentiated neurons, samples size N = 3 correspond to independent setup cultures, neuronal differentiation and analysis at different times. Exceptions where N = 2: i) Fig. 5, large volume of human cells and culture media were needed, but observations were validated across two independent and distinct cellular models, providing enough confidence on the conclusions; ii) Fig. 6, N= 2 but technical replicates were included (samples analyzed twice), and the results were also validated in another experiment (Fig. 8). Tau ELISAs, viability measurements and qRT-PCR (multi-well plate-based assays) include technical replicates per biological replicate. In experiments involving animals, N = 3 with further experimental details on sample (brain, plasma) generation and analysis are included in the Methods section.
Data exclusions	Pertaining to the compounds and cellular models that are the focus of this manuscript, no data was excluded from analysis.
Replication	All experimental findings were replicated at least 3 times with enough reproducibility. Attempts of data replication were, therefore, successful.
Randomization	Samples were allocated randomly for culture and analysis. For animal studies, allocation into experimental groups is not relevant because, in this study, we only describe proof-of-concept testing of one compound (AZD2014) in 3 animals, and measure drug brain penetration and plasma levels.
Blinding	In general, the investigators were blind at the time of experiment execution and data acquisition. For micro-dialysis and mass spec, analysis and quantitation were done blinded to group/sample allocation since samples were identified only by individual numbering without subdivision into groups. The pharmacokinetic study was run unblinded and all subsequent steps of data analysis were also unblinded. Determination of concentrations and PK parameters are considered as objective measures, not subject to bias and therefore the integrity of the results are not impacted when running the study and analysis unblinded.

Reporting for specific materials, systems and methods

We require information from authors about some types of materials, experimental systems and methods used in many studies. Here, indicate whether each material, system or method listed is relevant to your study. If you are not sure if a list item applies to your research, read the appropriate section before selecting a response.

Materials & experimental systems

n/a	Involved in the study	
☐	☒	Antibodies
☐	☒	Eukaryotic cell lines
☒	☐	Palaeontology
☐	☒	Animals and other organisms
☒	☐	Human research participants
☒	☐	Clinical data

Methods

n/a	Involved in the study	
☒	☐	ChIP-seq
☒	☐	Flow cytometry
☒	☐	MRI-based neuroimaging

Antibodies

Antibodies used	Western blot antibodies: TAU5, Invitrogen AHB0042. P-tau S396, Invitrogen 44752G. LC3, Cell Signaling Technology 3868, clone D11. LAMP2, Abcam ab18528. LAMP1, Cell Signaling Technology 9091, clone D2D11. p62, Enzo Lifesciences BML-PW9860. P-p62 S403, GeneTex GTX128171. ATG12, Cell Signaling Technology 2010. CTSD, Cell Signaling Technology 2284. mTOR, Cell Signaling Technology 2983T, clone 7C10. P-mTOR S2448, Cell Signaling Technology 5536T, clone D9C2. p70S6K, Cell Signaling Technology 9202. P-p70S6K T389, Cell Signaling Technology 9205S. S6, Cell Signaling Technology 2317, clone 54D2. P-S6 S240/244, Cell Signaling Technology 2215. 4E-BP1, Cell Signaling Technology 9644S. P-4E-BP1 T37/46, Cell Signaling Technology 9451S. BECN1, Cell Signaling Technology 4122S, clone 2A4. P-BECN1 S15, Cell Signaling Technology 84966S, clone D4B7R. AKT, Cell Signaling

Technology 9272. P-AKT S473, Cell Signaling Technology 4060, clone D9E. AMPKα, Cell Signaling Technology 5831, clone D5A2. P-AMPKα T172, Cell Signaling Technology 2535, clone 40H9. HSP90, Cell Signaling Technology 4877, clone C45G5. COX IV, Cell Signaling Technology 4850S, clone 3E11. HDAC2, Abcam ab7029. EEA1, Cell Signaling Technology 3288S, clone C45B10. RAB5, Cell Signaling Technology 2143S. CD9, Cell Signaling Technology 13403S, clone D3H4P. CD81, Santa Cruz sc-23962, clone 5A6. β-III-Tubulin, Sigma T-8660. GAPDH, Abcam ab8245, clone 6C5. β-Actin, Sigma A1978, clone AC-15.

IF antibodies: K9JA, Agilent A002401-2. PHF-1, Kindly provided by Dr. Peter Davies (Albert Einstein College of Medicine). MAP2, Millipore Sigma AB5543. Hoechst 33342, Thermo Fisher Scientific 62249.

Secondary antibodies: Anti-mouse IgG HRP-linked, Cell Signaling Technology 7076V. Anti-rabbit IgG HRP-linked, Cell Signaling Technology 7074S. Goat anti-chicken IgG Alexa Fluor 488, Life Technologies A11039. Goat anti-chicken IgY Alexa Fluo 594, Life Technologies A11042. Goat anti-rabbit IgG Alexa Fluor 594, Invitrogen A11012. Goat anti-mouse IgG Alexa Fluor 488, Invitrogen A11029. Goat anti-mouse IgG Alexa Fluor 594, Life Technologies A11032.

Validation

Tau antibodies were previously used (Silva et al. 2016 Stem Cell Reports, Silva, Ferguson et al 2019 eLIFE) and further validated in this study (Supplementary Fig. 2j, k).

-LC3 detects endogenous levels of total LC3B protein. Cross-reactivity may occur with other LC3 isoforms. Stronger reactivity is observed with the type II form of LC3B. Species Reactivity: Human, Mouse, Rat. Species predicted to react based on 100% sequence homology: Monkey, Bovine, Pig. Applications: WB, IHC, IF, Flow Cytometry,

-LAMP2 reacts with Mouse, Human, Monkey, Chinese hamster. Predicted to work with: Dog. Positive control WB: MEF1 whole cell lysate and mouse lung, human liver, and human liver (membrane fraction) tissue lysates. Suitable for: WB, ICC/IF, Flow Cyt, IHC-P.

-LAMP1 recognizes endogenous levels of total LAMP1 protein. Species Reactivity: Human, Monkey. Applications WB, IP, IHC, IF, Flow Cytometry.

-p62 Source: Purified from rabbit serum, Peptide affinity purified. Species reactivity: Human. Applications: ELISA, IF, IHC, WB.

-P-p62 (S403) IgG. Application WB, ICC/IF, IHC-P, FACS, IHC. Reactivity Human, Mouse, Rat.

ATG12 Antibody (Human Specific) detects endogenous levels of total free and Atg5 bound Atg12 protein. Species Reactivity: Human.

-Cathepsin D Antibody detects endogenous levels of preprocathepsin D, procathepsin D and the heavy chain subunit of mature cathepsin D. Species Reactivity: Human, Monkey. Application WB.

-mTOR rabbit detects endogenous levels of total mTOR protein. Species Reactivity: Human, Mouse, Rat, Monkey. Species predicted to react based on 100% sequence homology: Horse. Application WB.

-Phospho-mTOR (Ser2448) XP detects endogenous levels of mTOR protein only when phosphorylated at Ser2448. Species Reactivity: Human, Mouse, Rat, Monkey. Species predicted to react based on 100% sequence homology: Rat, Chicken, Pig, Horse. Application WB.

-p70 S6 Kinase Antibody detects endogenous levels of total p70 S6 kinase protein. This antibody also recognizes p85 S6 kinase. Species Reactivity: Human, Mouse, Rat, Monkey. Application WB.

-Phospho-p70 S6 Kinase (Thr389) Antibody detects endogenous levels of p70 S6 kinase only when phosphorylated at threonine 389. This antibody also detects p85 S6 kinase when phosphorylated at the analogous site (Thr412), and possibly S6KII phosphorylated at Thr388. Species Reactivity: Human, Mouse, Rat, Monkey. Application WB.

-S6 Ribosomal Protein (54D2) detects endogenous levels of total S6 ribosomal protein independent of phosphorylation. Species Reactivity: Human, Mouse, Rat, Monkey, D. melanogaster. Application: WB, IGC, IF, Flow Cytometry.

-Phospho-S6 Ribosomal Protein (Ser240/244) Antibody detects endogenous levels of ribosomal protein S6 only when phosphorylated at serines 240 and 244. This antibody does not detect S6 ribosomal protein phosphorylated at other sites. Species Reactivity: Human, Mouse, Rat, Monkey, Zebrafish. Species predicted to react based on 100% sequence homology: Chicken, Xenopus. Application WB and IP.

-4E-BP1 (53H11) detects endogenous levels of total 4E-BP1 protein. Species Reactivity: Human, Mouse, Rat, Monkey. Applications WB, IHC, IF, Flow Cytometry.

-Phospho-4E-BP1 (Ser65) Antibody detects endogenous levels of 4E-BP1 when phosphorylated at serine 65. This antibody has been shown to also recognize 4E-BP1 when phosphorylated at serine 101. Species Reactivity: Human, Mouse, Rat, Monkey. Application WB, IP.

-Beclin-1 (2A4) recognizes endogenous levels of total human Beclin-1 protein. Species Reactivity: Human. Application WB, IP.

-Phospho-Beclin-1 (Ser15) (D4B7R) recognizes transfected levels of Beclin-1 protein only when phosphorylated at Ser15 (which corresponds to Ser14 in mouse). Species Reactivity: Human, Mouse. Application WB.

-Akt Antibody detects endogenous levels of total Akt1, Akt2 and Akt3 proteins. The antibody does not cross-react with related kinases. Species Reactivity: Human, Mouse, Rat, Hamster, Monkey, Chicken, D. melanogaster, Bovine, Dog, Pig, Guinea Pig. Species predicted to react based on 100% sequence homology: Dog. Applications WB, IP, IF, Flow Cytometry.

-Phospho-Akt (Ser473) (D9E) XP detects endogenous levels of Akt only when phosphorylated at Ser473. Species Reactivity: Human, Mouse, Rat, Hamster, Monkey, D. melanogaster, Zebrafish, Bovine. Species predicted to react based on 100% sequence homology: Chicken, Xenopus, Dog, Pig. Application WB, IP, IHC, IF, Flow Cytometry.

-AMPKα (D5A2) detects endogenous levels of AMPKα protein. The antibody detects both the α1 and α2 isoforms of the catalytic subunit. Species Reactivity: Human, Mouse, Rat, Monkey, Bovine. Application WB, IP.

-Phospho-AMPKα (Thr172) (40H9) detects endogenous AMPKα only when phosphorylated at threonine 172. The antibody detects both α1 and α2 isoforms of the catalytic subunit, but does not detect the regulatory β or γ subunits. Species Reactivity: Human, Mouse, Rat, Hamster, Monkey, D. melanogaster, S. cerevisiae. Species predicted to react based on 100% sequence homology: Chicken, Zebrafish, Bovine, Pig. Application WB, IP, IHC.

-HSP90 (C45G5) detects endogenous levels of total HSP90 protein. This antibody does not cross-react with other HSPs. Species Reactivity: Human, Mouse, Rat, Monkey. Species predicted to react based on 100% sequence homology: Bovine. Application WB, IHC, IF, Flow Cytometry.

-COX IV (3E11) detects endogenous levels of total COX IV protein. Species Reactivity: Human, Rat, Monkey, Zebrafish, Bovine, Pig. Application WB, IP, IHC, IF, Flow Cytometry.

-HDAC2 ChIP Grade antibody. Tested and Suitable for: WB, IP, IHC-P, Dot blot, ICC/IF, ChIP. Species reactivity: Mouse, Human. Predicted to work with: Rat, Chicken.

-EEA1 (C45B10) detects endogenous levels of total EEA1 protein. Species Reactivity: Human, Mouse, Rat. Applications: WB, IF, IP.
Rab5A Antibody detects endogenous levels of total Rab5A protein. Species Reactivity: Human, Mouse, Rat, Hamster, Monkey. Applications: WB, IF.
-CD9 (D3H4P) recognizes endogenous levels of total CD9 protein. Species Reactivity: Human. Applications: WB, IHC.
-CD81 Antibody (5A6) is a mouse monoclonal IgG1 (kappa light chain) raised against OCI-LY8 cells. Recommended for detection of CD81 of mouse, rat and human origin by WB, IP, IF and FCM.
-β-III-Tubulin is derived from the SDL.3D10 hybridoma produced by the fusion of mouse myeloma cells and splenocytes from BALB/c mice. The antibody specifically recognizes an epitope located on isotype III of β-tubulin. No cross-reactivity with other tubulin isotypes is observed. The antibody can stimulate microtubule assembly when reconstituted with tubulin and either tau or MAP2 species reactivity: bovine, boar, rat, human. Applications WB, ICC, Flow Cytometry
-GAPDH antibody shows no cross-reaction with GAPDH from yeast. Tested applications: ELISA, ICC, WB, ICC/IF, IHC-Fr. Species reactivity: Mouse, Rat, Rabbit, Chicken, Hamster, Cat, Dog, Human, Pig, Xenopus laevis, Fish, Monkey, Zebrafish, Baboon, African green monkey. Predicted to work with: Horse, Guinea pig, Xenopus tropicalis. Does not react with: Goat, Cow, Saccharomyces cerevisiae.
-β-Actin antibody recognizes an epitope located on the N-terminal end of the β-isoform of actin. Species reactivity: pig, Hirudo medicinalis, bovine, rat, canine, feline, human, rabbit, carp, mouse, guinea pig, chicken, sheep. Applications: IHC, ICC, IF, WB, Microarray.
-MAP2 use in IC, IH & WB. It is expected that the antibody will react with all mammals.

Eukaryotic cell lines

Policy information about cell lines

Cell line source(s)	The neural progenitor cell (NPC) lines were generated in house (laboratory of Dr. Stephen J. Haggarty, PhD) from the respective human induced pluripotent stem cells (iPSC). This methodology has been previously published (Sheridan et al. 2011 PLoS One, Silva et al. 2016 Stem Cell Reports, Seo et al. 2017 J. Neuroscience, Cheng et al. 2017 Curr Protoc Hum Genet) and summarized in the Methods section.
Authentication	Cell lines were karyotyped after reprogramming, and NPCs were subjected to aCGH (Microarray-based Comparative Genomic Hybridization) analysis to confirm genomic stability. Tau encoding gene A152T variant, P301L mutation and wild-type were confirmed by SANGER sequencing. Standard iPSC and NPC quality control control procedures were followed (Silva et al. 2016 Stem Cell Reports, Seo et al. 2017 J. Neuroscience).
Mycoplasma contamination	All cell lines tested negative for Mycoplasma contamination.
Commonly misidentified lines (See ICLAC register)	No misidentified lines used.

Animals and other organisms

Policy information about studies involving animals; ARRIVE guidelines recommended for reporting animal research

Laboratory animals	Han Wistar male rats, age 8-10 weeks old.
Wild animals	No wild animals used.
Field-collected samples	None collected.
Ethics oversight	Animal studies were performed under Institutional Animal Care and Use Committee (IACUC)-approved protocol #PK-R-06012018. Statement also included in Methods section.

Note that full information on the approval of the study protocol must also be provided in the manuscript.
